# Discrepancies and potential impacts of self-reported versus measured height and weight on adult transthoracic echocardiography findings

**DOI:** 10.1186/s44156-025-00095-y

**Published:** 2025-11-10

**Authors:** Christopher Benson, David Austin, Richard Graham, Chris Wilkinson

**Affiliations:** 1 Academic Cardiovascular Unit, South Tees NHS Foundation Trust, Middlesbrough, UK; 2 Population Health Science Institute, Newcastle University, Newcastle upon Tyne, UK; 3 Hull York Medical School, University of York, York, UK; 4 Cardiac Investigations Unit, The James Cook University Hospital, Marton Road, Middlesbrough, UK

**Keywords:** Transthoracic echocardiography, Self-reported height, Self-reported weight, Estimated height, Estimated weight, Service evaluation, Body surface area

## Abstract

**Introduction:**

Accurate chamber quantification in transthoracic echocardiography (TTE) is important for guiding clinical decision-making. We aimed to assess the accuracy and reliability of patients’ self-reported height and weight compared to measured height and weight, and how any differences in the calculated body surface area (BSA) may affect TTE parameter classification.

**Methods:**

Consecutive patients attending for out-patient TTE were prospectively recruited at a large NHS Trust as part of a service evaluation. Height and weight were initially self-reported and then measured. TTE parameters were subsequently indexed to BSA or height based on both self-reported and measured values and compared.

**Results:**

698 patients participated. Self-reported and measured height, weight, and BSA were strongly correlated (*r* > 0.90). There was a difference between the mean self-reported and measured height (self-reported being 1.1 cm higher, *p* < 0.001) and weight (self-reported being 1.6 kg lower, *p* < 0.001) as well as the resulting BSA (self-reported being 0.01m^2^ lower, *p* = 0.008). Indexing TTE parameters to self-reported (rather than measured) values resulted in changes to the indexed left ventricular end-diastolic volume, left ventricular end-systolic volume, Sinuses of Valsalva diameter and proximal ascending aorta diameter (all *p* < 0.05), although the effect sizes were small.

**Conclusion:**

Compared to measured height, weight and calculated BSA, self-reported values are statistically different but result in little clinically important change to TTE parameters in out-patients attending for TTE. However, given the possible impact on clinical decision-making, TTE conclusions based on self-reported height and weight should be interpreted with care, particularly indexed left ventricular volumes and aorta dimensions. Echocardiographers should be vigilant in identifying rare cases where individuals significantly misreport their height or weight.

## Introduction

Transthoracic echocardiography (TTE) is the most widely used non-invasive cardiac imaging modality [1–3], and the quantification of cardiac chamber size by TTE commonly guides decision-making [2, 4–6]. Therefore the accurate and reproducible measurement of chamber size and comparison to a reference range is recommended in clinical guidelines [2, 4].

Whether a given TTE parameter is considered normal varies according to subject-specific characteristics including height, weight, ethnicity, gender, fitness and age [4]. A pragmatic approach is taken in clinical practice by indexing an individual’s TTE parameters to their height or calculated body surface area (BSA) [2, 4]. BSA is derived from a patient’s height and weight, which are commonly self-reported by the patient. However, the accuracy of self-reported height and weight in out-patients attending for TTE is unknown, as is the potential impact of self-reported values on indexed TTE parameters.

We aimed to quantify the difference between self-reported and measured height and weight and to examine the resulting differences in calculated BSA and indexed TTE parameters.

## Methods

Consecutive consenting out-patients attending for TTE between the 22nd March and 19th July 2024 were enrolled in this service evaluation.

### Setting

The James Cook University Hospital (JCUH) and the Friarage Hospital (FH) cardiorespiratory departments, South Tees NHS Foundation Trust.

### Participants

Out-patients attending the echocardiography department for TTE for indication were included. Patients attending community diagnostic centres and cardiology clinics were excluded for staffing reasons, as were patients attending during support worker industrial action (total 14 days).

### Variables

Patients were asked to report their height and weight (using imperial or metric measurements). They then had their height and weight measured (Marsden M-100 Column Weighing Scale). Their BSA was calculated by the study team using the Du Bois formula (BSA = 0.007184 x Height^0.725^ x Weight^0.425^) based on self-reported height and weight (‘*self-reported BSA’*) and measured height and weight (‘*measured BSA’*). The TTE parameters that are typically indexed (to either BSA or height) were then retrospectively extracted from authorised reports, including: left ventricular end diastolic volume (ml), left ventricular end systolic volume (ml), left ventricular mass (g), left atrial volume (ml), Sinuses of Valsalva diameter (mm), proximal ascending aorta diameter (mm), right ventricular end diastolic area (cm^2^) and right atrial area (cm^2^).

### Data sources and measurement

All TTEs were performed and reported by (or under the supervision of) British Society of Echocardiography (BSE) accredited echocardiographers (level II adult TTE) using GE (Vivid E95, Vivid S70) or Philips (Epiq, Affinity) echocardiography machines. Reports were retrospectively accessed using IntelliSpace Cardiovascular (Philips) reporting software to extract the required TTE parameters. These were then indexed to both the self-reported and measured BSA or height in accordance with the BSE guidelines: Sinuses of Valsalva diameter and proximal ascending aorta diameter were indexed to height, and the remaining parameters were indexed to BSA. Outcomes were categorised as normal or abnormal according to BSE reference ranges [4]. Where guidelines and reference ranges permitted, the degree of abnormality was also included (e.g. mild, moderate, severe).

### Statistical methods

All available data were included in the analysis of self-reported and measured height, weight and BSA. However, where self-reported or measured height and/or weight were unavailable, the participant was excluded from TTE parameter analysis as there was no comparator. After confirmation of normality of data distribution through histograms, skewness, and kurtosis values, frequencies and means with standard deviation (SD) were reported. Bland-Altman plots were generated to compare self-reported and measured height, weight and BSA. Pearson’s correlation coefficient was evaluated between self-reported and measured height, weight and BSA. Statistical significance and effect size between self-reported and measured height, weight and BSA were assessed with a paired T-test and Cohen’s d. A paired T-test was repeated to compare indexed TTE parameter values derived from self-reported and measured BSA or height. Significance was considered at 5%. A sub-analysis was performed to analyse the difference in self-reported versus measured height according to age.

### Ethics

In accordance with guidance from the Health Research Authority decision tool and discussions with the Research and Development department of South Tees NHS Foundation Trust, this work was pre-registered, approved, and conducted under a service evaluation framework. Each participant provided informed verbal consent to take part in this service evaluation.

## Results

### Participants

**Fig. 1:**
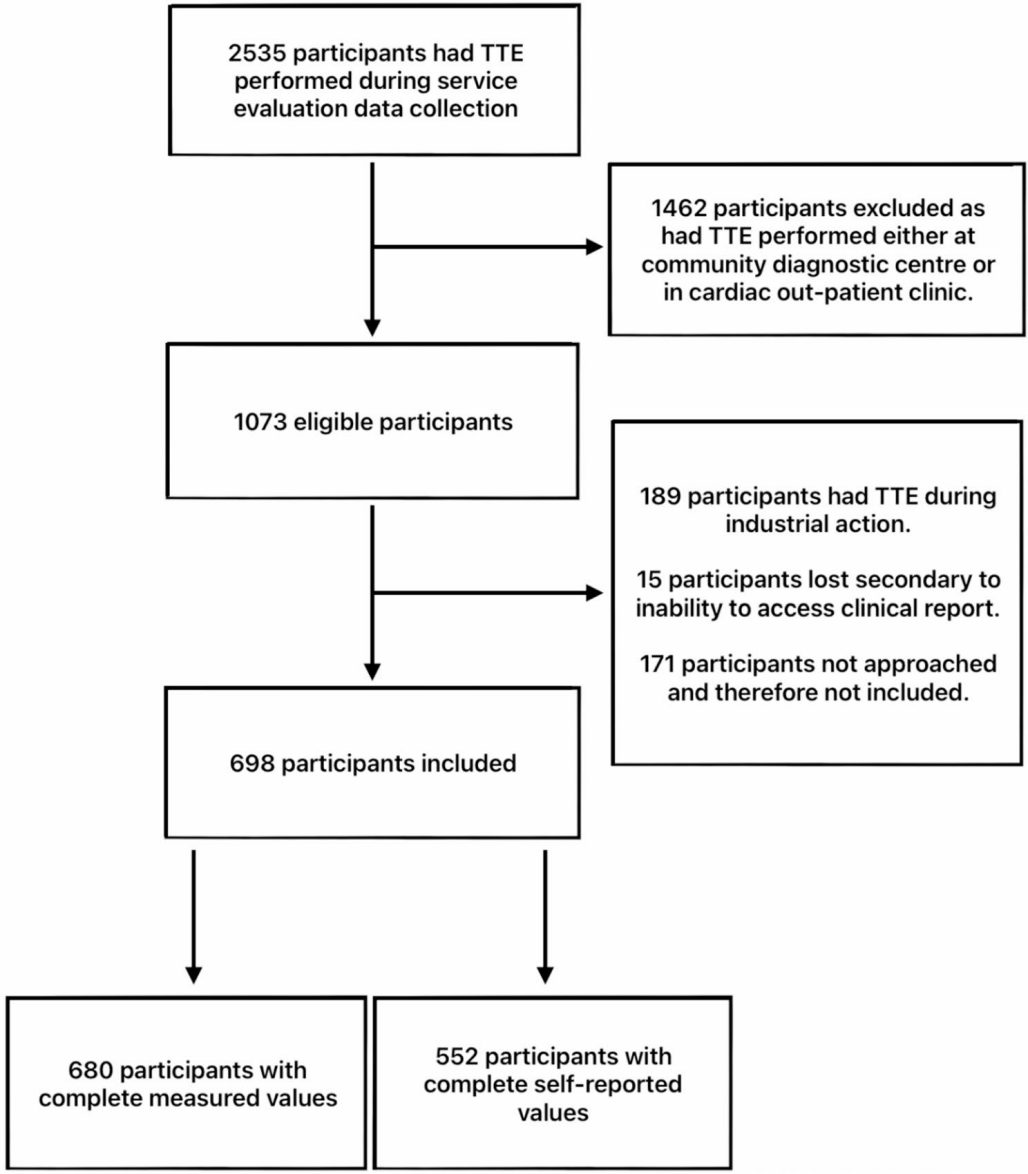
STROBE diagram of participants

In total, 698 patients participated from JCUH (*n* = 414) and FH (*n* = 284) (Fig. 1). There were 326 females (46.7%) and 372 males (53.3%), with a mean age of 65.7 (SD 16.8) years.

There were 146 participants (20.9%) who were unable to provide a self-reported height and/or weight and 18 (2.6%) whose height and/or weight could not be measured. By site, 38 participants (9.0%) at JCUH were unable to provide a self-reported height and/or weight, compared with 108 participants (38.1%) at FH. At JCUH, there were 14 participants (3.4%) for whom height and/or weight could not be measured, compared to 4 participants (1.4%) at FH, primarily due to frailty or being wheelchair-bound.

### Comparison of self-reported and measured height, weight and body surface area

**Fig. 2:**
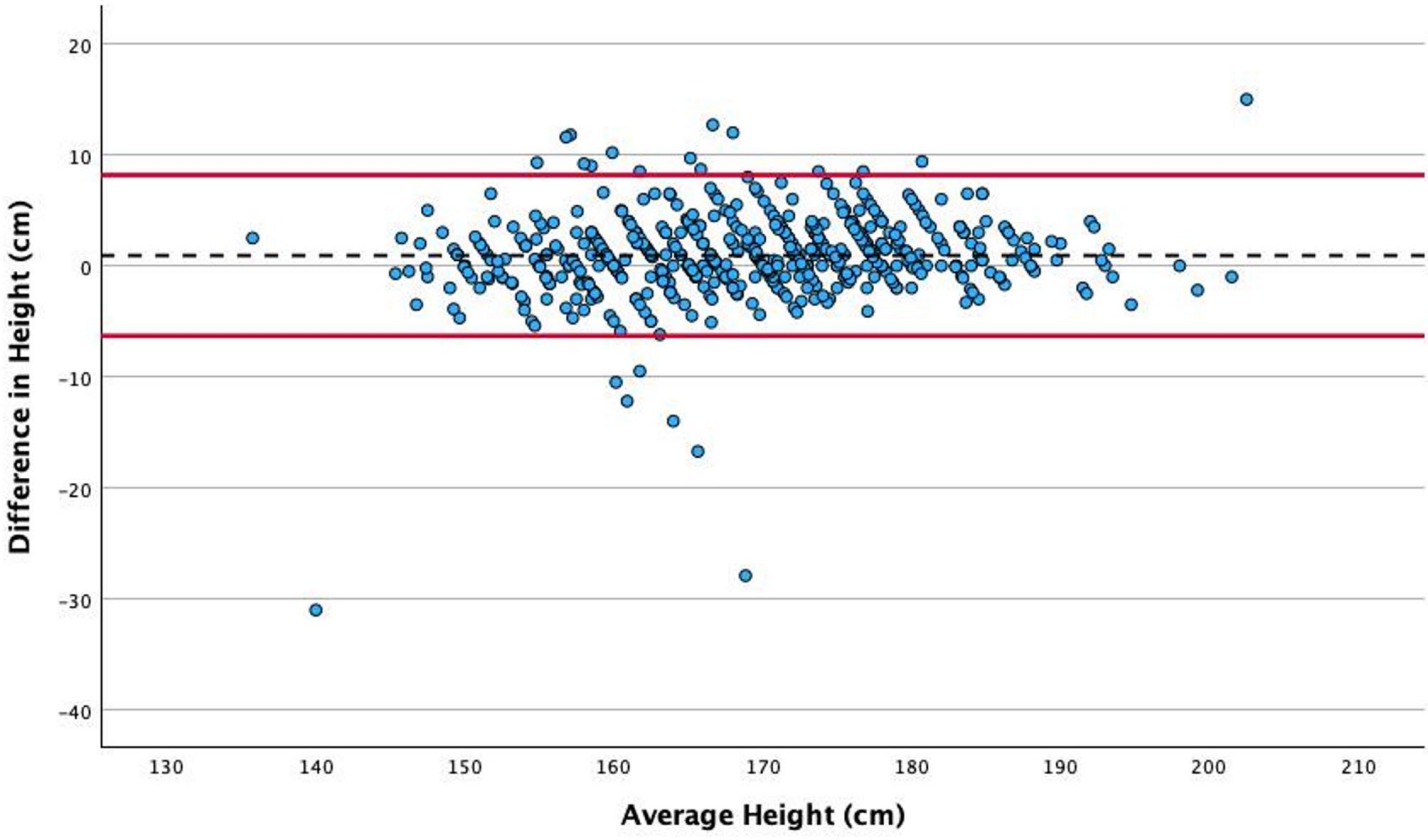
Association between self-reported and measured height. The black dotted line indicates the mean value, and the solid red lines indicate the upper and lower limits of agreement (+/- 1.96 SD from the overall mean difference)

**Fig. 3:**
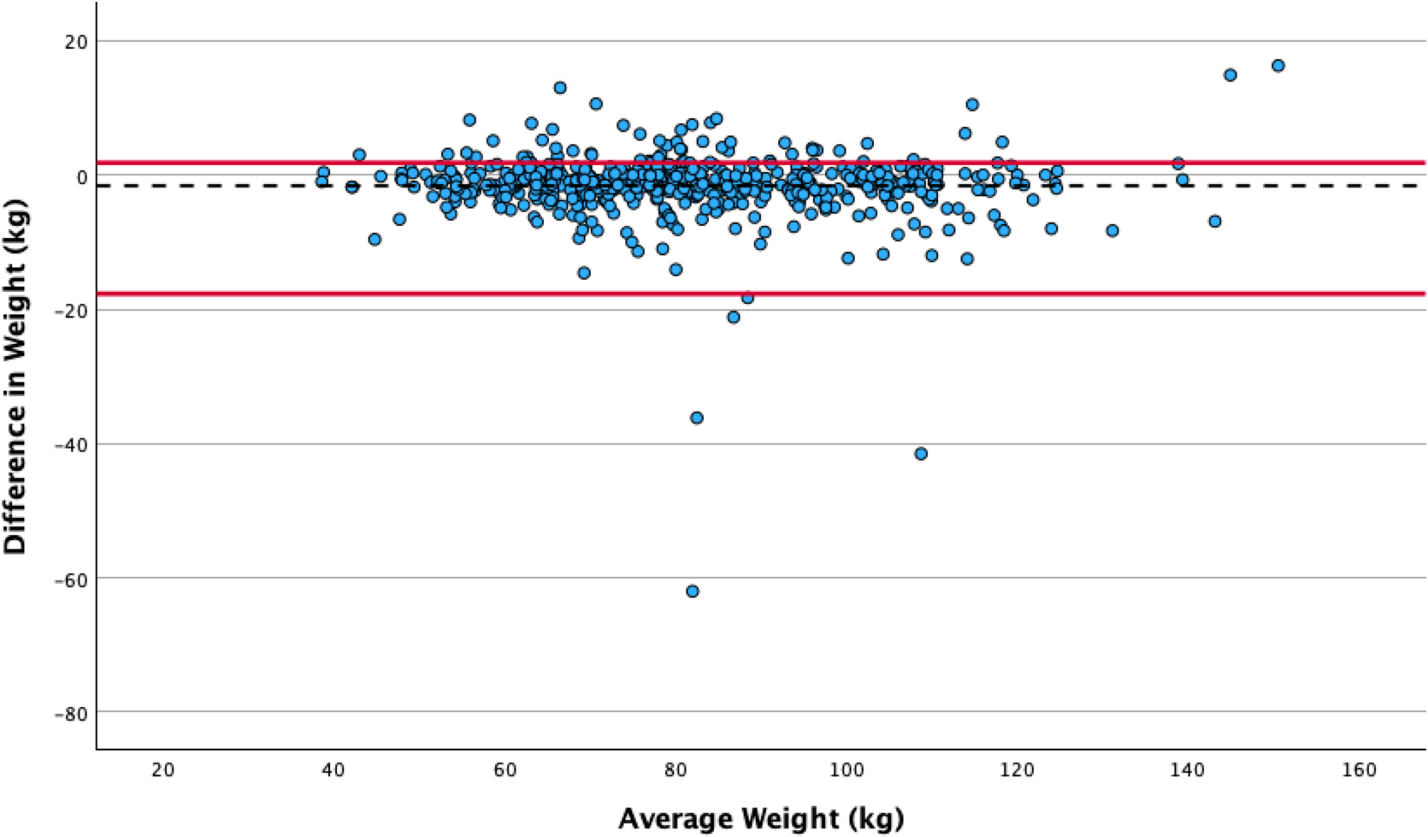
Association between self-reported and measured weight. The black dotted line indicates the mean value, and the solid red lines indicate the upper and lower limits of agreement (+/- 1.96 SD from the overall mean difference)

**Fig. 4:**
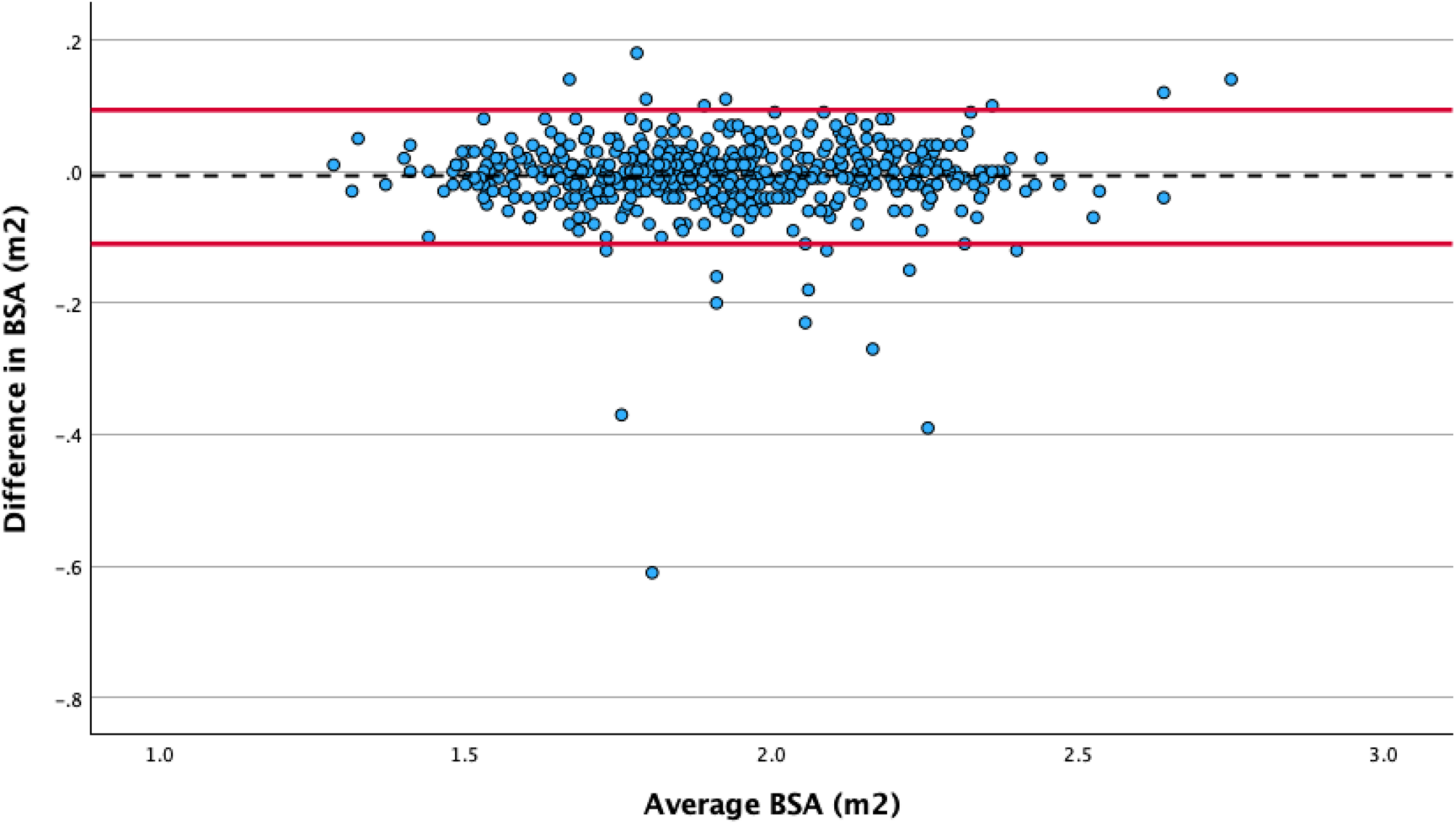
Association between self-reported and measured body surface area. The black dotted line indicates the mean value, and the solid red lines indicate the upper and lower limits of agreement (+/- 1.96 SD from the overall mean difference)

Self-reported and measured height (*r* = 0.94, *p* < 0.001), weight (*r* = 0.95, *p* < 0.001) and BSA (*r* = 0.97, *p* < 0.001) were strongly correlated (Figs. 2, 3 and 4). The mean self-reported height was 1.1 cm higher than the measured height (169.5 [SD 10.9] cm compared to 168.4 [SD 10.5] cm, *p* < 0.001). The mean self-reported weight was 1.6 kg lower than the measured weight (80.8 [SD 19.0] kg compared with 82.4 [SD 19.2]kg, *p* < 0.001), resulting in a derived mean BSA difference of 0.01m^2^ (self-reported 1.91 [SD 0.25] m^2^ compared with measured 1.92 [SD 0.25] m^2^, *p* = 0.008).

**Fig. 5:**
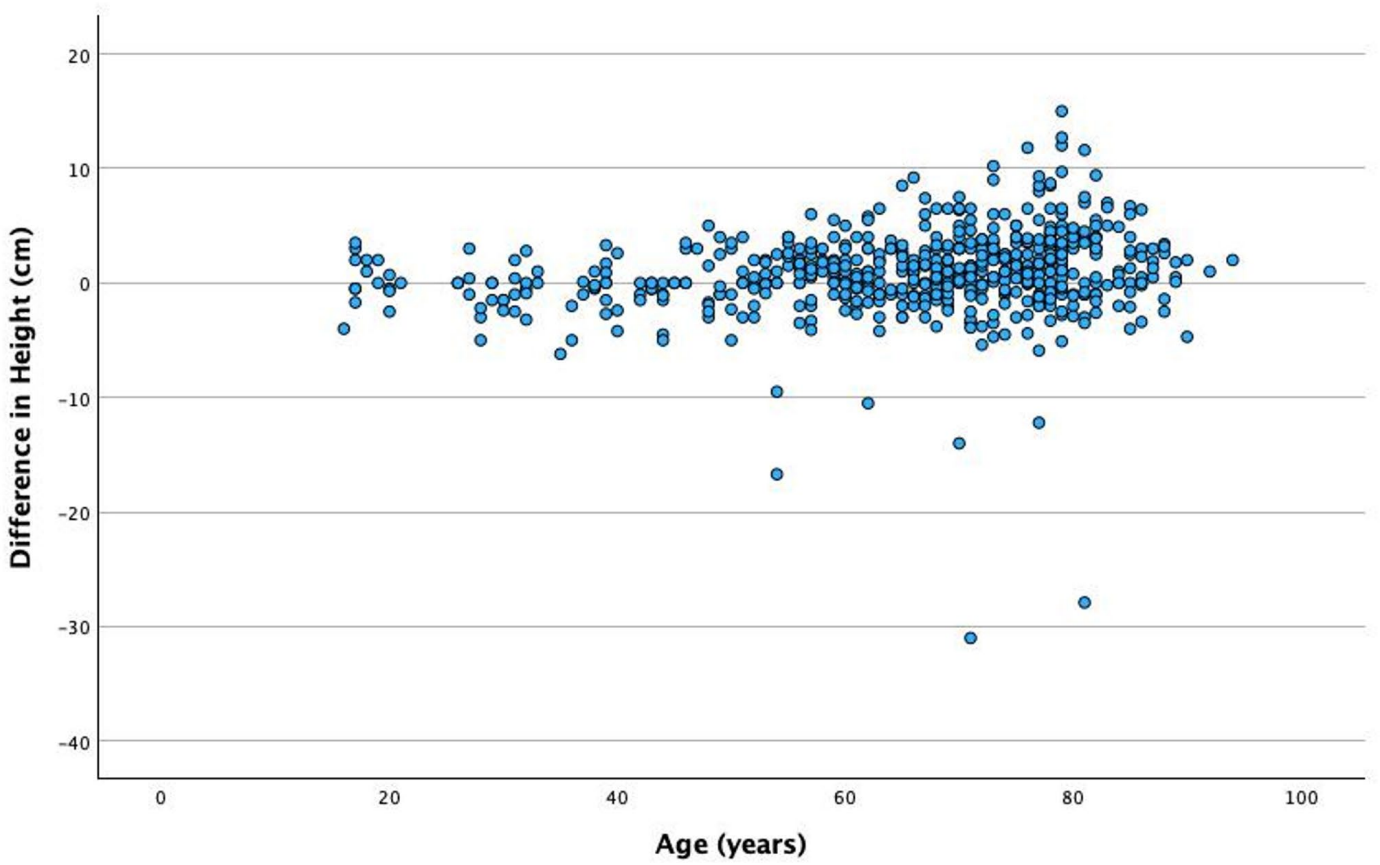
The difference between measured and self-reported height by age

**Fig. 6:**
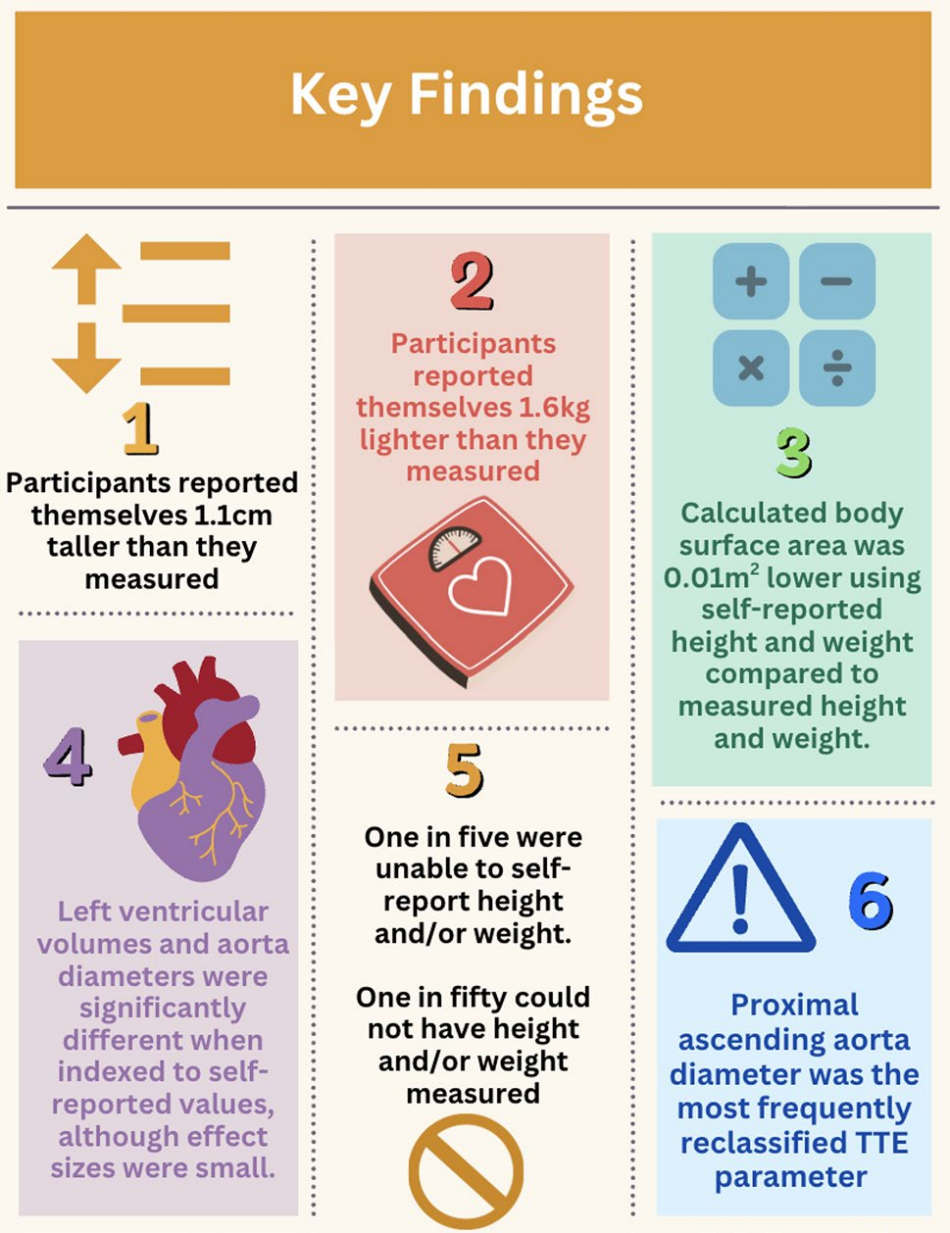
Key findings of this service evaluation

The sub-analysis of difference in self-reported and measured height according to age illustrates a tendency to over-report height between the ages of 60 and 80 years (Figs. 5 and 6).

### Comparison of indexed transthoracic echocardiography parameters

There were differences between the TTE parameters that were indexed to self-reported and measured BSA or height for left ventricular end-diastolic volume (49.3 [SD17.6] ml/m^2^ compared to 50.1 [SD 18.4] ml/m^2^, p = < 0.001; d = 0.25), left ventricular end-systolic volume (21.3 [SD 12.6] ml/m^2^ compared to 21.6 [SD 12.7] ml/m^2^, *p* = 0.006; d = 0.19), Sinuses of Valsalva diameter (18.6 [SD 2.5] mm/m compared to 19.0 [SD 5.6] mm/m, p = < 0.001; d=-0.29), and proximal ascending aorta diameter (18.4 [SD 3.0] mm/m compared to 18.6 [SD 3.2] mm/m, *p* < 0.001; d=-0.23)

There was no statistically significant difference in TTE parameters indexed to self-reported and measured BSA or height for left atrial volume (32.4 [SD 20.2] ml/m^2^ compared to 32.6 [SD 19.4] ml/m^2^, *p* = 0.079), right atrial area (3.4 [SD 1.6] cm^2^/m^2^ compared to 3.4 [SD 1.6] cm^2^/m^2^, *p* = 0.0.52), right ventricular end diastolic area (8.7 [SD 2.0] cm^2^/m^2^ compared to 8.6 [SD 2.1] cm^2^/m^2^, *p* = 0.019), and left ventricular mass (91.7 [SD 26.6] g/m^2^ compared to 91.7 [SD 27.2] g/m^2^, *p* = 0.049) (Table 1).

**Table 1:**
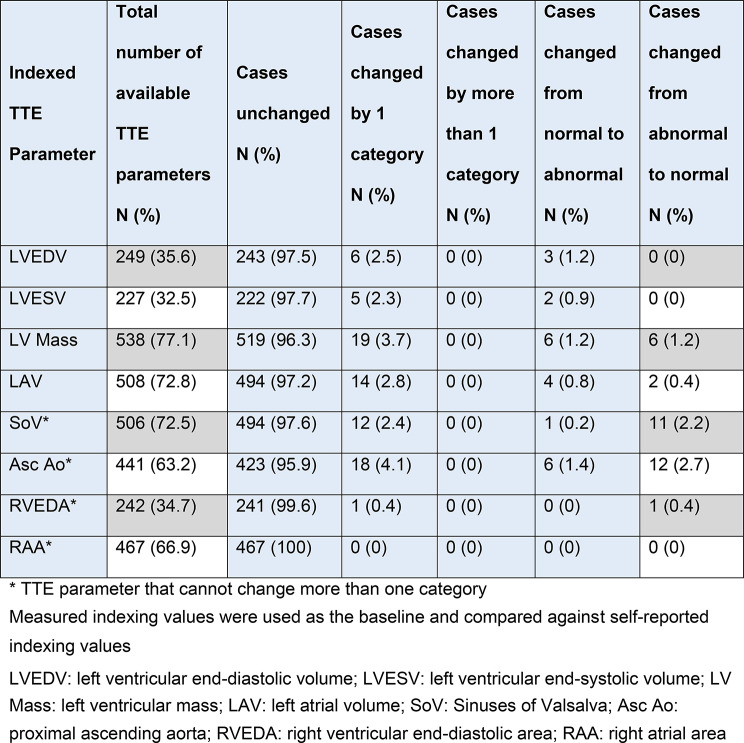
Change in TTE parameter indexed classification between self-reported and measured values

The number of cases with TTE parameters that remained unchanged by using self-reported versus measured values ranged from 95.9% (e.g. proximal ascending aorta 423/441) to 100% (e.g. right atrial area 467/467). The number of cases changed by one category (regardless of normality) across all TTE parameters ranged from 0% (e.g. right atrial area 0/467) to 4.1% (e.g. proximal ascending aorta 18/441). There were no cases that changed by more than one category. The number of cases that changed from normal to abnormal across all TTE parameters ranged from 0% (e.g. right ventricular end-diastolic area 0/242 and right atrial area 0/467) to 1.4% (e.g. proximal ascending aorta 6/441). The number of cases that changed from abnormal to normal across all TTE parameters ranged from 0% (e.g. right atrial area 0/467) to 2.7% (e.g. proximal ascending aorta 12/441). The number of cases across all TTE parameters that changed and crossed the normality threshold in either direction ranged from 0% (e.g. left ventricular end-diastolic volume 0/249, left ventricular and-systolic volume 0/227 and right atrial area 0/467) to 4.1% (proximal ascending aorta (18/441). The proximal ascending aorta diameter was the most likely TTE parameter to change classification and cross the normality threshold.

## Discussion

One in five patients were unable to provide a self-reported height or weight, and nearly one in fifty were unable to have height and weight measured. Of the participants where self-reported values were available, there was a small but statistically significant difference between self-reported and measured height, weight and BSA. This discrepancy resulted in variations in indexed left ventricular end-diastolic volume, left ventricular end-systolic volume, Sinuses of Valsalva diameter and proximal ascending aorta diameter. Across all TTE parameters up to 4.1% participants were reclassified when indexed to self-reported values compared to measured values.

### Accuracy and reliability of self-reported vs. measured height, weight and BSA

A meta-analysis by Wells and Goldstein compared height and weight estimation methods finding that self-reported height and weight were highly accurate and reliable techniques [9]. While this service evaluation did identify significant difference between self-reported and measured height and weight, the absolute difference, effect size and chance of clinical impact were all small. Furthermore, the discrepancies identified are comparable with the existing literature, in which the mean difference in height ranges from 0.5 to 1.3 cm and in weight from 1.1 to 1.5 kg [7, 8, 10]. In this study, we report a difference of 1.1 cm in height and 1.6 kg in weight. The absolute difference was smallest in BSA, which is explained by the overestimation of height and underestimation of weight combined with the Du Bois formula for BSA, which will act to mitigate the overall error. The tendency to over-report height and under-report weight is a common finding in the literature [7–10], particularly amongst those who are underweight or obese [7, 9, 10]. We did not observe this trend, which may relate to sample size or potentially participant awareness that their height and weight would be measured immediately after providing a self-reported height and weight, which may have encouraged more accurate estimates. Another possible explanation is differences in the specific populations that were studied, which were limited to adolescents, emergency care patients and women from the United States [7, 8, 10]. In contrast, this service evaluation included patients undergoing TTE in the North of England, who may have differing awareness of their current height and weight from these other populations.

### Comparison of indexed transthoracic echocardiography parameters

There were no TTE parameters that changed by more than one category, although in BSE guidelines, aortic diameters, right ventricular and right atrial areas have binary classifications and so cannot change by more than one category [4]. The proximal ascending aorta diameter was the most commonly observed TTE parameter to cross the normality threshold from abnormal to normal. This is a result of aortic diameters being indexed to height only. This may be explained by a bias among participants aged 60 years or older to over-report their height and could reflect recall of their lifetime peak height, as opposed to current height, which will have reduced over time with normal ageing processes. This may mean that using measured indexing values for aortic diameters could create increased demand for echocardiographic aorta surveillance in patients who are arguably being misrepresented as abnormal secondary to normal age-related height loss. To this end, departments could consider formulating local consensus statements to prevent the overburdening of resource-limited services. It is not known whether current height or lifetime peak height is most relevant regarding cardiac and cardiovascular dimensions. However, currently, there is no recommended alternative to indexing TTE parameters to height and BSA.

### Feasibility of self-reported and measured height, weight and BSA

In one in five cases, participants were unable to offer a self-reported height and/or weight. This figure is higher than in a previous study, where 12.4% of participants were unable to self-report height and/or weight [11]. The authors found that elderly, overweight, and female participants were more likely to be unable to self-report their weight [11]. In our study, there was a larger proportion of participants unable to self-report height and or weight at FH compared to JCUH. This difference may stem from inconsistencies in data collection processes. At JCUH, self-reported height and weight were collected in a clinical room. In contrast, FH lacked a suitable clinical space, requiring these measurements to be taken in a corridor, away from other patients. Although efforts were made to ensure privacy, patients might have felt uneasy sharing their height and weight in a public space. As a result, they may have opted to have the healthcare professional measure this information and record it without verbalisation.

A true lack of awareness regarding one’s height and weight is also a recognised phenomenon, as highlighted by Lipsky and Haynie [12], who documented that between 0.2% and 1.4% of participants provided biologically implausible height and/or weight estimates. This is evident in this service evaluation where one subject had a difference of more than 30kg between their self-reported and measured weight, one had a difference greater than 40kg, and one greater than 60kg. Similarly, for self-reported and measured height, four individuals showed a discrepancy exceeding 10cm, one individual had a difference greater than 20cm, and another exceeded 30cm. In each of these cases, self-reported height, weight and BSA should not be used as they would result in substantial errors in TTE parameter classification. These participants were included in data analysis to best represent real-world data. It can be argued that the preservation of these results may have skewed the overall findings and produced substantial differences that are not observable in the majority of cases. Nevertheless, the small absolute mean differences in height, weight, and resulting BSA demonstrate an overall minimal impact of these cases, which, despite their significance, are numerically minor.

### Body surface area

There is debate surrounding the suitability of BSA as an indexing parameter for TTE. This mainly concerns the validity of BSA formulae, especially for obese individuals, alternative indexing parameters, and notable differences among various BSA formulae [13–18]. Nevertheless, BSE and the American Society of Echocardiography / European Association of Cardiovascular Imaging (ASE/EACVI) guidelines recommend using BSA for indexing measurements of left ventricular and left atrial volumes, left ventricular mass, and right ventricular and right atrial areas. Variations exist between guidelines, for example, ASE/EACVI recommend indexing aorta dimensions to BSA, while BSE recommends indexing to height [2, 4]. Furthermore, the European Society of Cardiology recommend that left atrial volume be indexed to height [19]. Obese patients present a problematic subgroup of patients regarding indexing TTE parameters to BSA, which can result in misinterpretation of the indexed parameter. This is especially important because obese patients are more likely to have diastolic dysfunction [20] and are concurrently more prone to having their indexed left atrial function inaccurately portrayed as normal due to inflated ‘normal ranges,’ thus, diastolic dysfunction can be missed if careful interpretation of indexed values is not performed. Foreshortened views may result in similar errors. Consequently, it is the responsibility of the echocardiographer to ensure that physiological data is best represented through non-foreshortened views. Equally, it is the responsibility of the referring clinician to pragmatically interpret indexed TTE parameters. A potential solution to this may be to use BSA derived from measured height and ‘ideal weight’, although further research is required in this area. It must also be recognised that while indexing can offer value in identifying patients likely to have ‘abnormal’ aorta dimensions, the indications for intervention are defined by absolute values only [21]. This scenario is similar to left ventricular diameter, where BSE argues that absolute measurements are predictors of outcomes and suggests no longer providing indexed measurements in reports [4].

### Impact of findings

Height and weight should be measured for every TTE patient. This is the gold standard to quickly calculate BSA and is guideline recommended [2, 4]. Moreover, this service evaluation supports using measured height and weight, highlighting that only one in fifty participants could not have their height and/or weight measured, compared to one in five who were unable to provide a self-reported height and/or weight. If height and weight measurements cannot be performed, our findings suggest that out-patients attending for TTE can generally self-report height and weight reliably and accurately, with a few notable exceptions that are likely to be apparent to the echocardiographer.

### Strengths and limitations

To the best of our knowledge, this is the first service evaluation to assess the discrepancies of self-reported height, weight and BSA to measured values and identify how any discrepancies may alter TTE parameter classification. This was a prospective study conducted at two sites (tertiary and secondary centres) that included a diverse range of participants. However, recruitment during the interval was negatively impacted by industrial action, and not all potential participants were approached to participate, which was likely to be due to competing demands for the clinical team, but may have introduced bias. This could have been mitigated by asking the echocardiographer to record height and weight, but this would have an impact on patient flow and echocardiographer time. Participants were weighed while wearing light clothes, which may explain their under-reporting of weight. However, no reasonable alterations could resolve this issue, and this represents routine clinical practice.

## Conclusion

In most cases, self-reported height and weight are sufficient for accurate calculation of BSA for clinical reports, although height is typically over-reported and weight under-reported. One in five participants were unable to provide self-reported height and/or weight, compared to one in fifty participants who could not have their height and/or weight measured. TTE conclusions can be drawn from self-reported indexed values, but diligence should be exercised regarding indexed left ventricular volumes and aorta dimensions. Professionals should be aware of rare instances where a significant discrepancy exists between self-reported and measured height and/or weight.

## Data Availability

The data that support the findings of this study are not openly available due to local collection and storage. Data is available from the corresponding author upon reasonable request.
